# ODM Data Analysis—A tool for the automatic validation, monitoring and generation of generic descriptive statistics of patient data

**DOI:** 10.1371/journal.pone.0199242

**Published:** 2018-06-22

**Authors:** Tobias Johannes Brix, Philipp Bruland, Saad Sarfraz, Jan Ernsting, Philipp Neuhaus, Michael Storck, Justin Doods, Sonja Ständer, Martin Dugas

**Affiliations:** 1 Institute of Medical Informatics, University of Münster, Münster, Germany; 2 Competence Center Chronic Pruritus, Department of Dermatology, University of Münster, Münster, Germany; University of Adelaide School of Medicine, AUSTRALIA

## Abstract

**Introduction:**

A required step for presenting results of clinical studies is the declaration of participants demographic and baseline characteristics as claimed by the FDAAA 801. The common workflow to accomplish this task is to export the clinical data from the used electronic data capture system and import it into statistical software like SAS software or IBM SPSS. This software requires trained users, who have to implement the analysis individually for each item. These expenditures may become an obstacle for small studies. Objective of this work is to design, implement and evaluate an open source application, called ODM Data Analysis, for the semi-automatic analysis of clinical study data.

**Methods:**

The system requires clinical data in the CDISC Operational Data Model format. After uploading the file, its syntax and data type conformity of the collected data is validated. The completeness of the study data is determined and basic statistics, including illustrative charts for each item, are generated. Datasets from four clinical studies have been used to evaluate the application’s performance and functionality.

**Results:**

The system is implemented as an open source web application (available at https://odmanalysis.uni-muenster.de) and also provided as Docker image which enables an easy distribution and installation on local systems. Study data is only stored in the application as long as the calculations are performed which is compliant with data protection endeavors. Analysis times are below half an hour, even for larger studies with over 6000 subjects.

**Discussion:**

Medical experts have ensured the usefulness of this application to grant an overview of their collected study data for monitoring purposes and to generate descriptive statistics without further user interaction. The semi-automatic analysis has its limitations and cannot replace the complex analysis of statisticians, but it can be used as a starting point for their examination and reporting.

## Introduction

Promoting new therapies or pharmaceutical products, randomized clinical studies are the means of choice to determine their utility. Clinical study data is mostly gathered on *case report forms* (CRFs), which are questionnaires consisting of multiple questions, so-called *items*, filled out by the physicians and study nurses. Although some studies are still performed on paper or based on spreadsheet applications like Microsoft Excel, more and more studies are using *electronic data capture systems* (EDCs) [1]; not only from a cost perspective [2]. Within EDCs, data is captured on electronic CRFs allowing direct validation, auditing and accessing of remotely entered data. In order to access trial data, EDC solutions—even open source or free-to-use software—are generally capable of exporting clinical data in the XML-based CDISC (Clinical Data Interchange Standards Consortium) ODM (Operational Data Model) format [3–6]. ODM is a standard to communicate and archive metadata as well as subject data [7, 8] of a study. Also for investigator initiated trials or smaller studies, EDC systems are advisable compared to spreadsheet based solutions due to security, traceability and quality reasons [9]. Commonly used commercial examples of EDC software are MACRO by Elsevier [10] or Medidata Rave [11], also open source solutions like OpenClinica [12] or REDCap [13] are existing. An example video of how to export an ODM file from OpenClinica is included as supplemental material, see S1 Video.

In clinical studies it is common practice to continuously evaluate already collected data. During the runtime of the study the clinical data is usually monitored focusing on CRF completeness and participant counts. Solutions for these monitoring tasks are often included in the EDCs themselves. At the end of a study, however, descriptive statistics of certain data items are required to present the study’s result. The CONSORT statement [14] recommends under point 15 to add baseline characteristics to any reports of clinical studies. These characteristics are also claimed by FDA in the FDAAA 801 [15] and are the main contribution of published *cohort profiles* [16]. The generation of these descriptive statistics is not supported by EDCs in general.

The usual workflow to generate these statistics is to import the EDCs’ data into statistical software such as SAS software [17], IBM SPSS [18], or R [19]. These tools are specially tailored to create complex statistics and even to create illustrative charts. However, since R is a statistical programming language, it cannot be easily applied by physicians and is often not suitable for simple tasks like creating item counts or generating descriptive statistics. In this case, a trained programmer is needed for the evaluation task. The other two tools, SAS software and IBM SPSS, provide, besides the programming interface, a graphical interface which allows the creation of descriptive statistics with only a little bit of training. But these tools are commercial and costly. SPSS for example costs in its basic configuration about 100 EUR per user per month [18]. Independent of the statistical software of choice, the applied statistical algorithm has to be manually configured for each item to analyze. This is a very time consuming process and thus increases the labor costs of the study’s stakeholders.

The objective of this work is to design, implement and evaluate a software package named ODM Data Analysis (ODM-DA), which addresses the previously described lack of statistical analysis in EDCs by providing freely available, easy to use software to generate tables of descriptive statistics. By uploading the clinical data in the ODM format, its syntax is validated, the completeness of the collected data is determined, and the descriptive statistics and additional charts of nearly all items are generated automatically. No further pre-setting or configurations need to be done and the results are clearly presented to the user.

## Materials and methods

### CDISC Operational Data Model

**Fig 1:**
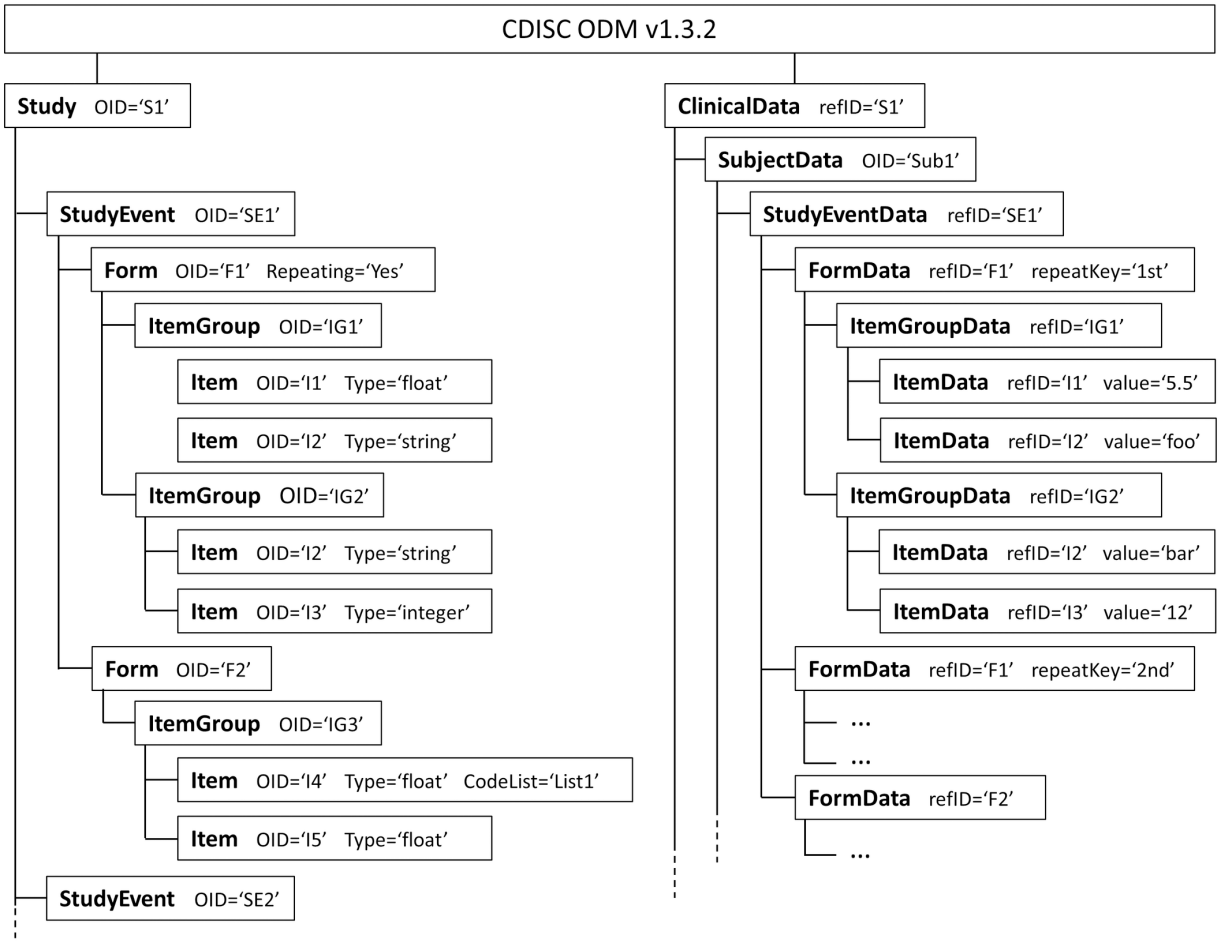
Schematic structure of an ODM file. Hierarchical structure of the ODM’s metadata on the left side and clinical data on the right side. The added attributes should clarify the connection between metadata and clinical data elements.

CDISC ODM [4] was chosen as input format since it is a common format exportable by most EDC systems. ODM fosters the standardization of clinical study metadata as well as clinical subject data. In the following the structure of ODM is described, since it is important for the understanding of certain design and interface decisions. The simplified structure of an example ODM file is illustrated in Fig 1.

ODM is divided into the definition of metadata (Study), i.e., the structure of the clinical studies CRF, and the clinical data (ClinicalData) itself, which is collected during the study. Although the ODM standard allows storing multiple clinical studies in a single ODM file, we assume only one study per file in this work for simplification and since it is the most common case. The metadata definition is structured hierarchically with a *Study* element on the first level. A Study may consist of multiple *StudyEvents*; for example *Baseline* or *Follow-Up* screenings during a study. Similar, each study event may contain several *Forms* which are equivalent to questionnaires. Each *Item*, i.e., question, is grouped in *ItemGroups* to provide a more clear form structure. Items are defined by data type, e.g., *float* or *string*, a question text and optional description, and may be associated with a *CodeList*, i.e., a selection of predefined options.

Each metadata element (*StudyEvent*, *ItemGroup*, *Form*, etc.) has a meaningful *name* and is identified by a locally unique identifier, so-called *OID*. Locally unique means, that the OID is unique within one ODM file so that an element can be present and reused in multiple arms of the hierarchy without the need of redefining the same item several times. For instance, each item can be included in multiple item groups, but only once inside each group.

The clinical data is shaped around subjects, i.e., participants of the study, which are represented by *SubjectData* elements and are distinguishable by a unique identifier. The structure itself mimics the metadata hierarchy and references each level’s element by its OID. On the item level, the collected value of the associated subject is stored.

In the clinical data, item groups, forms, and study events can be contained multiple times for a single subject, if flagged as repeating in the metadata. This feature is used to handle recurring events, which number of appearances is unknown at the beginning of the study. An example is a *serious adverse event* form, which may occur several times for a patient during a study or does not occur at all. In this case, these elements are distinguished by a *RepeatKey* which is used as a local discriminator.

### Validation of the input file

To enable the automatic generation of statistics, certain requirements must be fulfilled by the input file. First of all, the syntax must be ODM standard conform to allow the automatic parsing of the file. This check is performed by validating the ODM file against the *XML schema definition* (XSD) provided by CDISC. Currently, ODM-DA supports the ODM versions 1.3.0, 1.3.1, and 1.3.2. Furthermore the ODM file must not contain any further XML extensions. If the file fails this check, further analyses are aborted, since no correct file parsing can be guaranteed.

If the file passes the syntactical validation, it does not imply the correctness or usefulness of the contained data. The XSD only checks the order and appearances of the XML tags in the ODM. It further checks the syntactical correctness of the attributes’ values, but not their semantical conformity. Thus, in a second validation process, the content is validated. This time, it will be checked if all references in the clinical data have been defined in the metadata part and if the clinical data items’ values are of the correct data type. We define a correct data type as a successful conversion from the XML value string into the data type defined by the meta item definition and, if a code list is associated with the item, the presence of the value in that list. If errors in the attributes are detected during this process, the analysis is not aborted as in the previous check, but the invalid clinical data entry is excluded from further analyses. All errors are displayed to enable the user to correct and re-upload the file.

### Provided statistics

Since we assumed only one study per ODM file, the first hierarchical metadata level taken into account is the level of study events. For each event the total number of references in the clinical data and the number of subjects which contribute to this event are calculated. Note, that the number of references may be larger than the number of subjects due to repeat keys.

The same computations are applied to the remaining hierarchical levels, i.e., forms, item groups, and items. It is important to note that all metadata elements are distinguished according to their position in the metadata tree. For instance, forms that are referenced in two study events will be treated as two separate forms during the calculations.

Furthermore, on the lowest metadata level, i.e., items, dependent on their data types, different statistics and charts are computed. We group the data types into five categories: dichotomous, nominal, ordinal, interval, and ratio. This distinction is oriented on the scale of measure by S. Stevens [20]. An overview of supported data types belonging to each category and their associated statistics is given in Table 1. The ODM standard supports more rare types, like partial dates, which are not implemented yet. In the following, the five categories are described in detail.

**Table 1:** Table illustrating the five different categories the application distinguishes and their calculated statistics and charts.

	ODM data types	Provided chart	Example statistical output
dichotomous	Boolean	Pie chart	True: 55 - False: 45
nominal	string, text	No chart	Diversity: 22 - Top3: 1. red (9) | 2. green (7) | 3. blue (5)
ordinal	CodeList	Bar chart	Diversity: 3/3 - Top3: 1. Yes (8) | 2. No (4) | 3. Maybe (3)
interval	time, date, datetime	Histogram	Range: 13:54:47 on 05 May 1920 - 20:11:59 on 09 June 2010
ratio	integer, float, double	Histogram	Min: 1.1 | Max: 8.8 | Mean: 4.9 | Median: 4.4 | StdDev: 2.6

#### Category: Dichotomous

This category only contains the ODM data type *Boolean*. Valid data values are the strings true/false or the numeric values 1/0. The quantity of these values, i.e., numbers of true and false, is calculated as statistic and a pie chart is used as graphical representation.

#### Category: Nominal

This category contains the character-based data types of ODM which are *string* and *text*. In ODM, there is no difference between these two types since *text* is a deprecated feature to support downwards compatibility. Since these items are based on free text input, it is hard to provide useful descriptive statistics in a general manner. Especially, if the item contains long text like a medical report. However, physicians tend to copy and paste to reduce the amount of needed documentation time [21]. Thus, we have chosen to calculate the top three exact matching strings for each item. String values may contain single concepts but also whole sentences, which are not further pre-processed before the comparison. The appearances of these strings are counted in a list and the top three values are provided to the user. For this kind of data no useful chart can be provided since an unknown number of different values can occur. If the number of different values is pre-defined, i.e., due to code lists in ODM, which can have character-based data types, a useful chart calculation is possible. However, we combine all code lists in the next category: ordinal.

#### Category: Ordinal

This category contains all items which are based on *CodeLists*, i.e., the clinical data can only contain a well-known number of different values defined by the associated list. In this case we do not distinguish the list’s data type which can be character-based or a numeric value. As statistic, like in the previous category, the number of appearances in the clinical data per option is counted and the top three options are provided to the user with their number of occurrences. Since code lists can become very huge, e.g., providing a complete *International Classification of Diseases* (ICD) list with more than 10.000 codes [22], the statistics are limited to the top three appearances. However, a bar plot is used showing at most ten list’s options. At most means, if the list contains ten or less different options, all options with their quantities are illustrated. If the list contains more options, then the top nine are displayed with their quantities and the last bar is used to summarize the quantities of the remaining options together, titled ‘others’.

#### Category: Interval

This category is used for the ODM data types *time*, *date* and *datetime*. Only the range values, e.g. the min and max, are provided as statistical output. Since these items often relate to the date of birth or the current date of a visit, this range value seems to be the most useful one. Thus, the duration of a study or the age distribution can be found out quickly. Providing an average value would provide no useful knowledge in the general case. However, the distribution of the different values inside this range is illustrated using a histogram.

#### Category: Ratio

This category contains all numerical ODM data types which are *integer*, *float* and *double*. For these data types the minimum and maximum value, median, mean and standard deviation are calculated. Like the previous category, a histogram is used to illustrate the distribution of all values. Note, that all values are treated internally as doubles for the chart calculation. Thus, the histogram’s bucket borders will be decimals even for integer values.

### Data completeness

Although this measure is often included in the EDCs themselves, our application can determine it as well, to provide a reference of how expressive the calculated statistics are and the clinical data itself is. Two different measures of completeness can be calculated. The first one is based on the mandatory flag in ODM. Each metadata element can be flagged as mandatory, which is defined in the ODM Standard as:

*The mandatory flag indicates that the clinical data for an instance of the containing parent metadata element would be incomplete without an instance of this type of element. ODM clinical data files that are incomplete in this sense may be considered incomplete for study review and analysis purposes*. [4]

For example, if a form contains an item group *adverse events* and an item *date of event* in this group, which is flagged as mandatory. The item group itself is not flagged as mandatory, since not every subject may have adverse events. Following the definition of mandatory, the date item does not have to be completed for each subject to accomplish completeness (as the term mandatory may imply). But, if the item group is present in the clinical data of a subject, then the date item must be completed. If the clinical data contains the item group 5 times, then the date item must be present also 5 times. If it is only present twice, our measure will calculate the completeness of the mandatory item in this case as 40% completed, and similarly 2 out of 5 item groups are completed. This calculation can be done recursively till the level of subjects is reached. Thus, the user gets an overview, how many subjects contain completed data and where data is missing in the hierarchy. However, this calculation requires a meaningful usage of the mandatory flags in the ODM file.

The second completeness measure that is provided is more general and can be applied if the mandatory flags are not set correctly. In this case, we are calculating the same measure but assuming each metadata element to be mandatory. Thus, the user gets feedback of the completeness of each metadata element. However, this measure will often show incomplete items if they are optional in the study such as conditional items, which are only filled out if a specific condition is fulfilled. Thus, this completeness must be interpreted with care.

### Implementation of ODM Data Analysis

We decided to implement ODM Data Analysis (ODM-DA) as a web application which enables physicians to use the service from their local web browser by only providing a single server. However, sending sensitive clinical study data—even if it is pseudonymized—to be temporarily processed on a foreign server in the internet is always a data protection risk. Therefore, ODM-DA is also available as Docker [23] image which can be downloaded from the Docker repository [24] to run the tool on a local PC or to start an own server for the organization in its local network. Thus, it can be controlled that the sensitive data is not leaving the organization.

ODM-DA is implemented in Java using the Spring framework [25] and the Bootstrap library [26], while Apache Tomcat [27] has been used as servlet container. Charts are generated with the JFreeCharts library [28] and a complete PDF report is created using the Apache PDFBox library [29]. According to the possible huge amount of Java objects that might be instantiated during the ODM parsing, we decided to import all elements into a H2 in-memory database [30]. H2 is used for performant calculations on the dataset.

### Datasets for evaluation

The performance of the tool has been evaluated with study data from four local registries. The MPADR registry (DS1) is a biobank to gain new knowledge about the development and course of arteriosclerotic manifestation [31]. The TBI registry (DS2) is aiming to set up and establish a cross-sectoral documentation of patients with traumatic brain injury [32]. The Pruritus Database (DS3) contains medical history of patients with chronic pruritus to define and compare different patient subgroups [33]. The CAD-REF registry (DS4) comprises data of patients with angiographically determined coronary artery disease and renal failure to develop models for identifying high-risk subgroups [34]. Size and contained elements of each dataset can be seen in Table 2. Each dataset has been uploaded five times into ODM-DA and the average time for the analysis process has been taken.

**Table 2:** Table of dataset characteristics used for the benchmark.

Dataset	File size	#StudyEvents	#Forms	#ItemGroups	#Items	#Subjects
DS1	10 MB	1	2	20	145	307
DS2	65 MB	1	13	111	658	1325
DS3	247 MB	6	6	60	369	6070
DS4	302 MB	7	19	89	873	3351

Each dataset is listed with their ODM file memory size and their contained quantity of elements.

Since the processing time, besides the dataset size, is dependent on the hardware configuration, we have performed the benchmarks on three different setups. The first configuration is a local execution of the program on a Linux desktop PC with 16 GB RAM, an Intel i5 and a maximal Java heap size of 8 GB. The second one uses the provided Docker image on the same PC. The last configuration uses the Docker image on a Windows 7 PC with the same hardware configuration, but is executing in a Linux virtual machine started by Docker, since Windows 7 does not support Docker natively.

## Results

### Workflow

**Fig 2:**
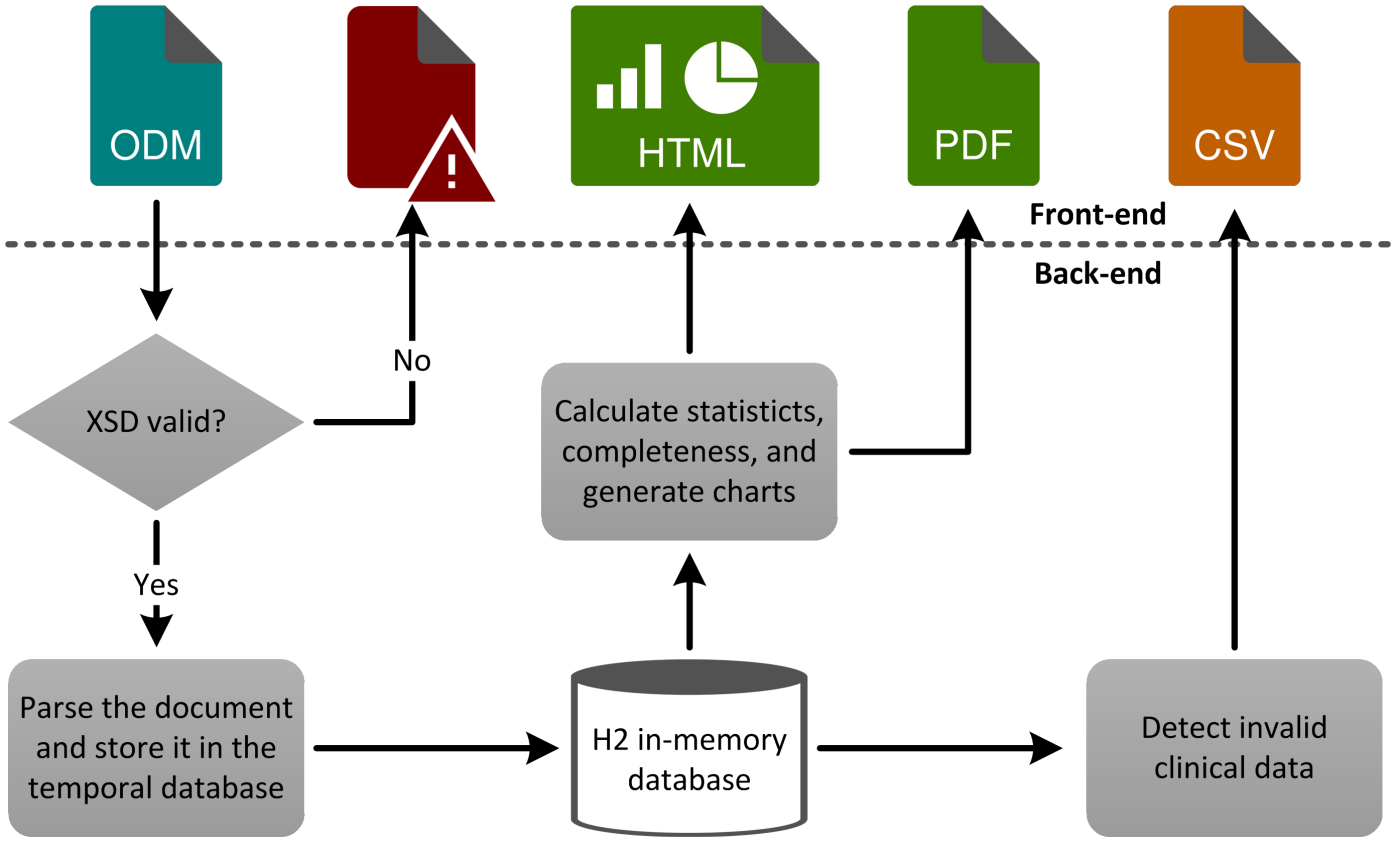
Schematic overview of the application’s workflow. The user can upload an ODM file via the web-based front-end. After parsing the file, its content is temporarily stored in a database. The calculated statistics and charts are presented on the result pages and are also generated as PDF. The PDF can be downloaded via the front-end and will be deleted from the server, equally the database content, after the session ends.

The general application’s workflow is illustrated in Fig 2. After uploading an ODM file to the application, the XML syntax is validated as described in the methods section. If errors are found, the process is terminated and a list of found syntactical errors is presented to the user.

In case of success the document is parsed and the contained metadata, e.g., forms and items, as well as the clinical data are inserted into the H2 in-memory database. The database is used for intermediate calculations like groupings and the reduction of the required Java heap space. During this process, syntactically invalid clinical data is detected and excluded from further processing. A list of all invalid data entries is provided to the user similar to the syntactical errors. The list of errors can be exported as CSV file to enable improvement of data quality by later manual corrections of invalid values.

Depending on the item’s data type, different descriptive statistics are calculated on the CPU as well as on the database itself. These statistics and charts are embedded in Java server pages and presented to the user, the same applies for the completeness calculations. A PDF report containing the entire analysis and all charts is created if initially selected. Each page of the PDF corresponds to a single item from the ODM file and can be downloaded for further examination.

### Interface

For a high user satisfaction and usability of ODM-DA, the challenge lies within the handling of large forms with hundreds of items, to provide an intuitive way for the user to navigate quickly to the items of interest. The main user interface of ODM-DA consists of two pages namely ‘Upload’ and ‘About’. As soon as a file is uploaded, a new ‘Results’ tab is added to the panel. Users can switch back to the ‘Upload’ page and upload another file anytime. The old results remain on the result page until the new upload has been started. The ‘Results’ page consists of sub-tabs, which depend on options checked on main page while uploading a file. It can consist of up to four sub-tabs namely Analysis, Completeness, Export PDF and Invalid Values. Thus, the user has the opportunity to adapt the file accordingly. The following explains the layout and purpose of all pages.

#### Cover page

**Fig 3:**
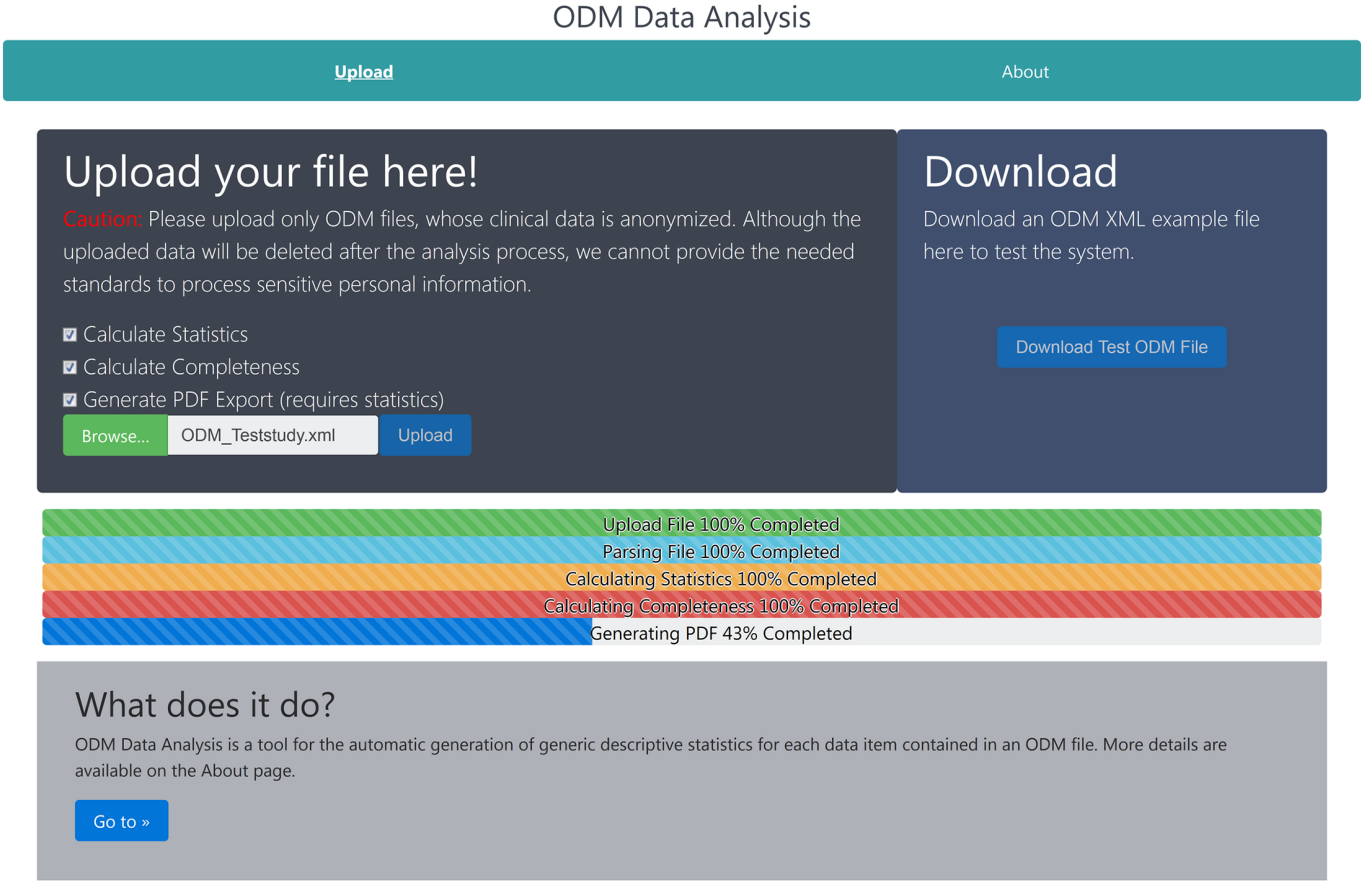
Cover page of the web-application. The image shows the first page presented to the user after starting ODM-DA. Besides allowing the upload of an ODM file for the analysis, the download of a test file and a link to the user manual are provided. In addition, the different analysis options can be specified.

After starting the application using the Docker image or visiting the web server, the cover page is presented to the user as shown in Fig 3. Here, a sample ODM file can be downloaded to test the application without the need of generating an ODM file. The test file showcases the statistics of all supported data types and illustrates some corner cases of the analysis, e.g., empty forms. The main purpose of the cover page is to upload any ODM file, select the desired analysis options, and to start the analysis. The current options are calculating statistics, calculating completeness and generating the PDF which requires the statistics calculation. During the analysis process, progress bars indicate the remaining analysis time. For each task, i.e., file upload, file parsing, statistics calculation, completeness calculation, and PDF creation, a separate progress bar is used. During the upload, the user can explore the rest of the website, for example the user manual on the about page, while the upload is being performed in the background.

#### Analysis page

**Fig 4:**
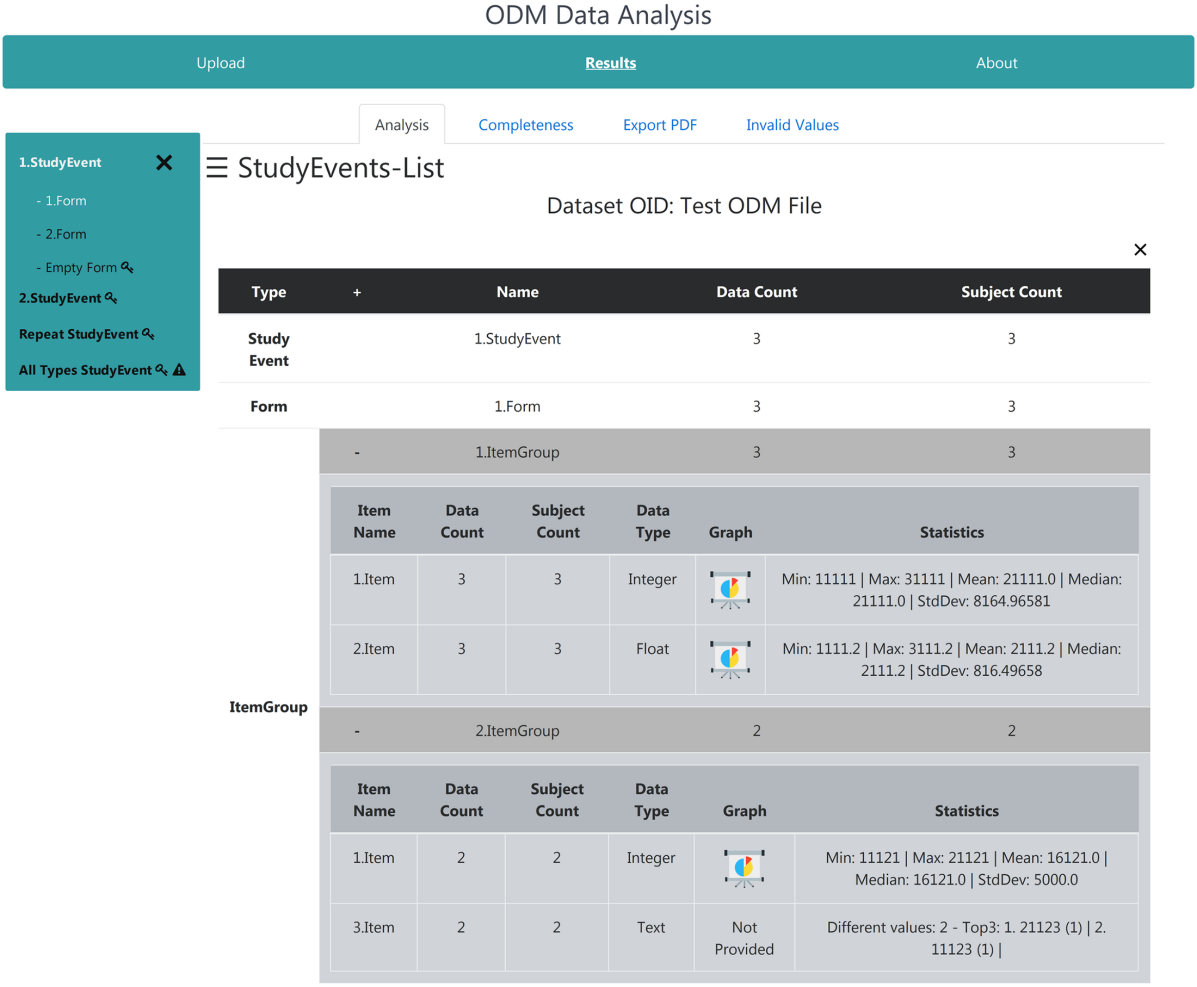
Analysis page of the web-application. Result page after uploading the provided test ODM file. The left navigation bar can be used to select forms from all study events contained in the clinical study data. The selected forms are shown on the right side, where each item group can be expanded and collapsed. By clicking the presentation icon, the associated chart of the item is shown. The key icons indicate the existence of repeat keys while the warn signs indicate invalid values.

After successful upload and validation with ‘Calculate Statistics’ enabled, the generated statistics are provided to the user as shown in Fig 4 on sub-tab ‘Analysis’. Here, the left navigation bar can be used to switch between study events and their associated forms in a tree like structure. The presence of repeat keys is highlighted by a small key icon next to the metadata names. If invalid clinical data is found, it is highlighted by a small warn sign next to the associated metadata elements. If a form is selected, it is visualized in the right table view. Multiple forms can be selected at the same time and will be visualized below each other. They can be closed again by re-selecting them in the left navigation bar or by clicking the x in the top right corner. General statistics of the form and study event are displayed in the top two rows of each selected form, i.e., their appearances in the clinical data and the distributing subject count. All contained item groups, with their statistics, are depicted in darker gray below the forms. To handle large forms, all item groups are initially minimized. By clicking a group, it extends and the statistics of its associated items are shown. By clicking the pie chart icon, the corresponding chart of the item is unfolded in the table by adjusting its position while resizing table’s cells. For faster navigation all item groups can be extended and minimized by clicking the ‘+’ symbol, which changes to ‘−’ when clicked and can be used to minimize all item groups at once, in the form header.

#### Completeness page

**Fig 5:**
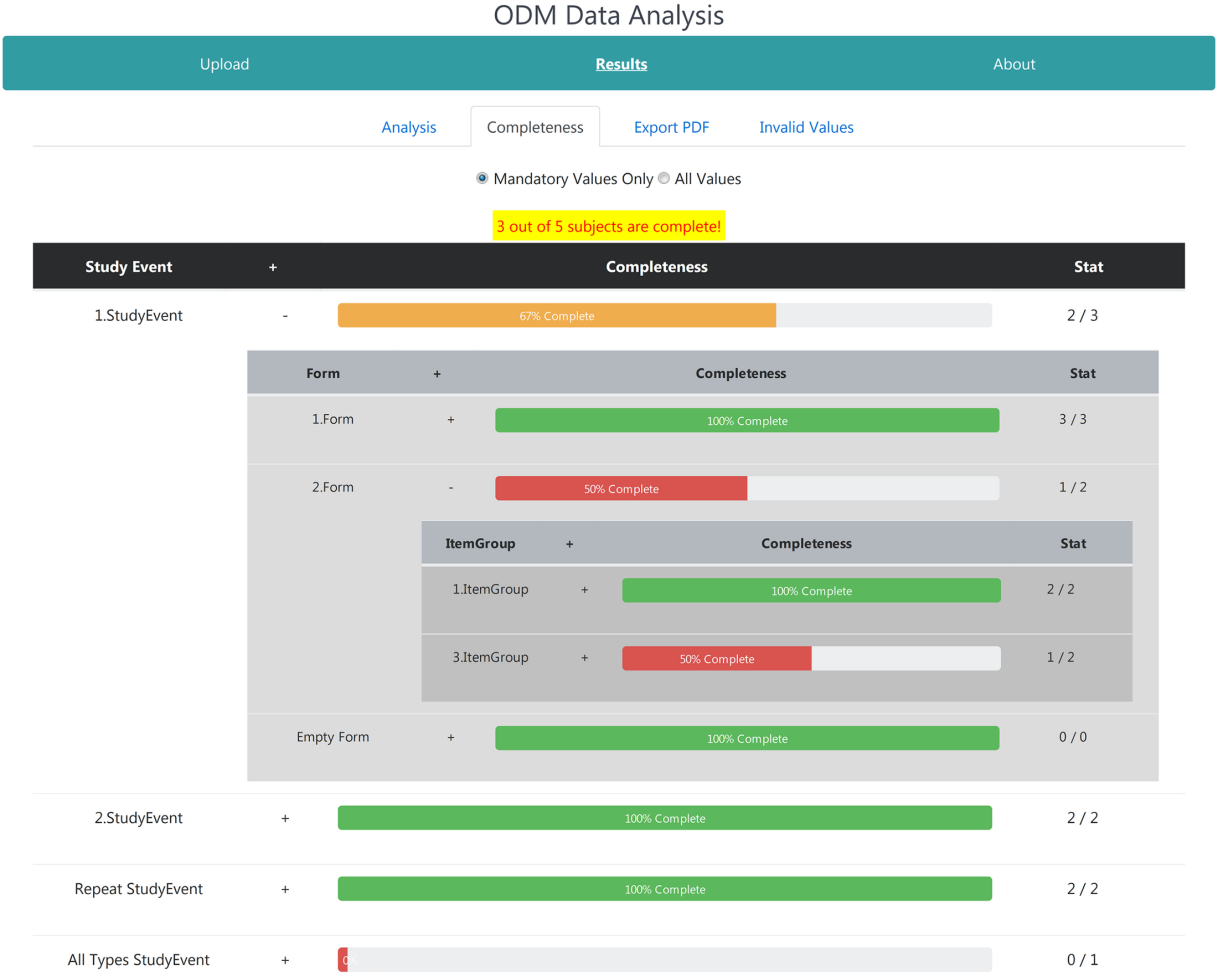
Completeness page of the web-application. Calculated completeness of the study data, i.e., if all items have been completed for each subject. The hierarchical structure of the metadata is displayed similar to the analysis page. Colored bars indicate the completeness of each metadata element.

If calculation of the completeness was selected on the cover page, the completeness tab is displayed to the user as shown in Fig 5. Similar to the analysis page, the different hierarchies of the metadata structure can be unfolded in a tree-like view. For each metadata element, a completeness bar with absolute values is presented. Its color encodes the percentage of the completeness and acts as quick visual feedback. The number of completed subjects is highlighted on the top of table in yellow color. The user can switch between both completeness options as described in the methods section.

#### PDF page

**Fig 6:**
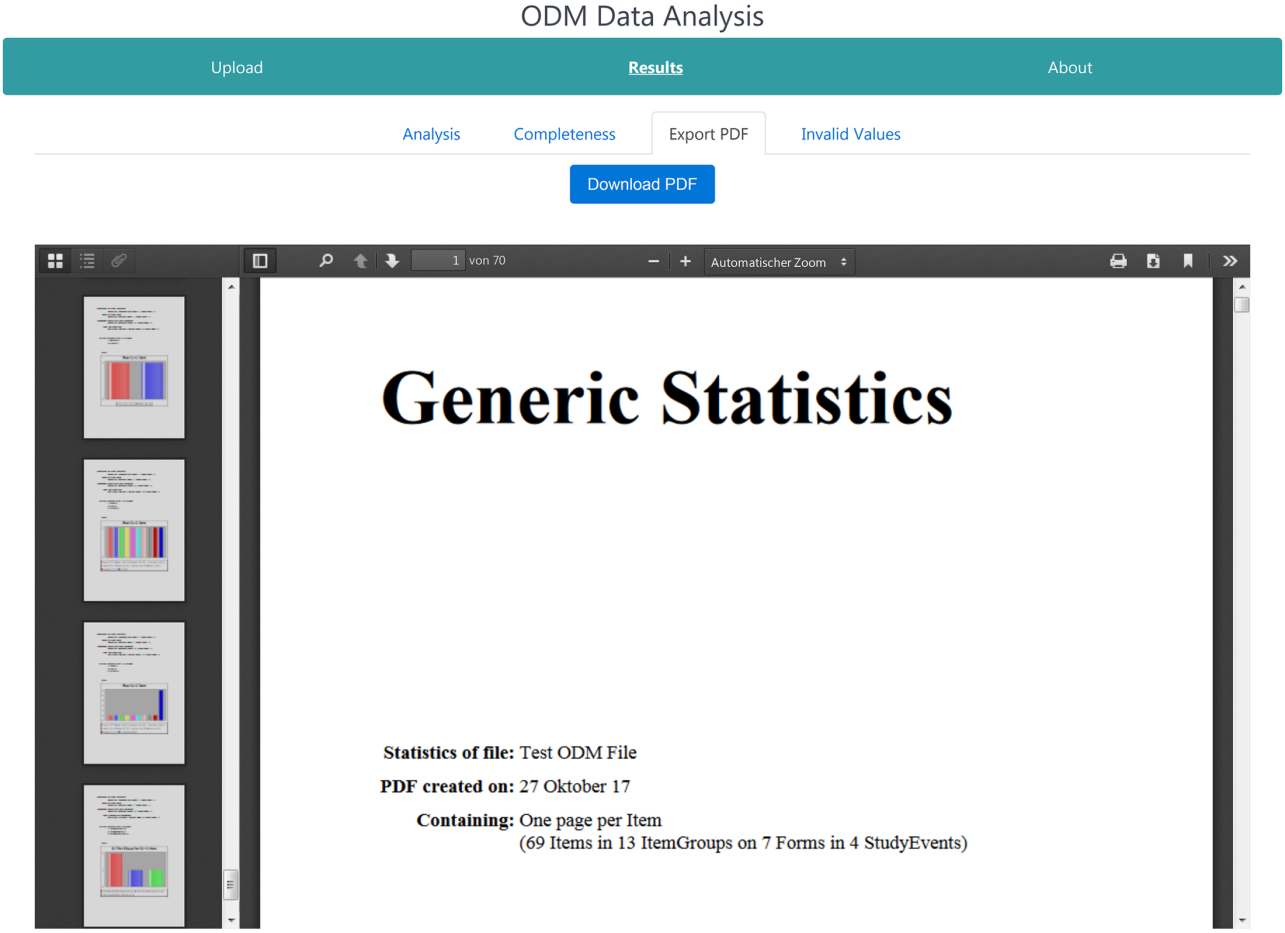
PDF page of the web-application. This page consists of a PDF viewer to view the generated PDF in the browser. It contains all calculated statistics and can be downloaded for later usage.

If the PDF export option was selected on the main page, the PDF tab is displayed to the user as shown in Fig 6. The generated PDF is opened in the web viewer, containing all charts and statistics. It can be downloaded from this page for further analyses or later usage by clicking the blue button on the top of the page.

#### Invalid value page

**Fig 7:**
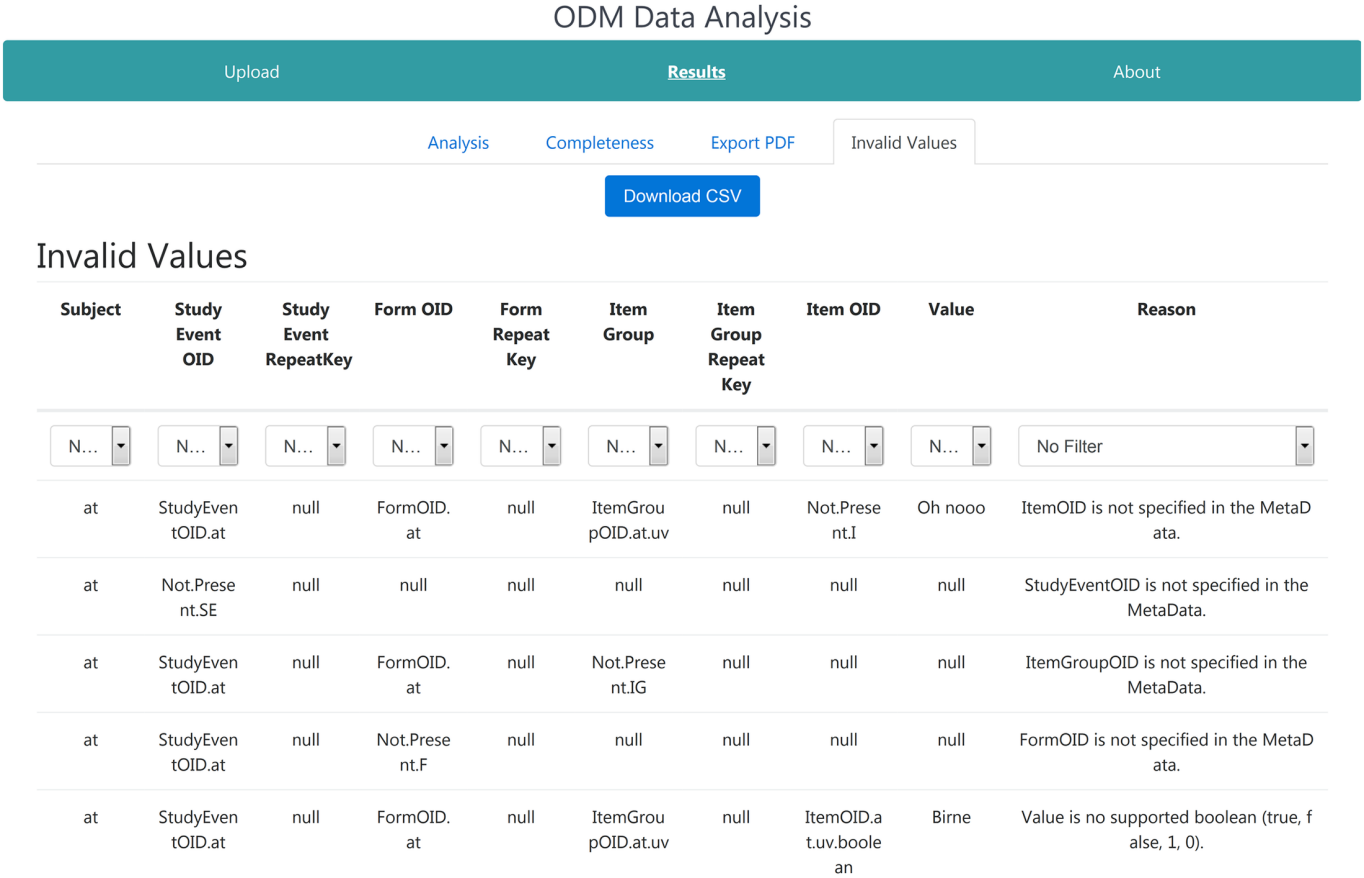
Invalid value page of the web-application. This page shows all detected invalid clinical data entries in the ODM file. For each error the entire path of the invalid entry in the hierarchical clinical data structure and the reason why it was considered invalid is displayed. The list can be filtered for each characteristic and exported as CSV file.

If the ODM file contains invalid clinical data, i.e., data elements without associated metadata definitions or a value of wrong data type, this tab is displayed to the user as shown in Fig 7. Here, for each detected error the path in the clinical data hierarchy is shown, including repeat keys, with the invalid entry reason. Filters can be used to filter the list for specific reasons or for certain OIDs. All detected errors can be exported as CSV file by clicking the ‘Download CSV’ button on top of the page.

### Performance benchmarks

All processing times of the benchmarks can be found in Table 3. Depending on the datasets size the processing time is increasing linearly. Generating the statistics takes equally much time as creating the PDF report. Overall, the time for the entire process is quite similar with the local Linux machine and the Docker instance. The Docker version on Windows 7 consumes sparsely more time.

**Table 3:** Table of performance benchmarks.

	Dataset	Parsing	Statistics	Completeness	PDF creation	Over all
Local (Linux)	DS1	10 s	1 s	7 s	3 s	21 s
	DS2	54 s	5 s	55 s	14 s	128 s
	DS3	272 s	36 s	378 s	8 s	695 s
	DS4	428 s	60 s	633 s	18 s	1139 s
Docker (Linux)	DS1	9 s	1 s	7 s	3 s	20 s
	DS2	57 s	6 s	55 s	15 s	133 s
	DS3	263 s	33 s	368 s	8 s	673 s
	DS4	474 s	62 s	652 s	20 s	1188 s
Docker (Windows 7)	DS1	10 s	1 s	8 s	3 s	22 s
	DS2	87 s	7 s	57 s	17 s	168 s
	DS3	430 s	49 s	427 s	8 s	914 s
	DS4	715 s	86 s	705 s	30 s	1536 s

The benchmarks have been performed for four datasets on three different setups, i.e., a local host, the Docker image on Linux and Windows 7. The average values of five benchmarks are listed.

## Discussion

The ODM-DA software is a simple to install and run web-application for rapid providing of automatically generated descriptive statistics and completeness analyses. Different access options (server, Docker instance) are provided to perform clinical research data analysis based on the ODM format. Data collection in clinical studies is increasingly performed by reputable EDC solutions offering different advantages [1, 35–38] and nearly every vendor offers exporting capabilities in ODM-XML [3]. In order to facilitate the process of generating basic statistics needed for reporting baseline data in clinical research—such as in the CAD-REF registry [34] -, there is no need to install software on a local PC, but rather we offer a web-application everyone can use in a secure network.

With ODM-DA generic statistics of nearly every ODM file can be generated for further analysis. Anyhow, the presented benchmark results of Table 3 must be considered with care. Processing times are highly dependent on factors such as current RAM usage, caching status, additionally running processes as well as Java thread and garbage collector handling. Nonetheless, the results show that even for large clinical studies the overall runtime is below 30 minutes.

By inspecting the benchmark results it appears, that for each of the three configurations the processing time increases linearly according to the datasets’ sizes. Only dataset 4 requires significantly more time, probably caused by the internal Java memory management. The time needed for the statistics and completeness calculations is significantly influenced by two parameters: The first one is the number of item tags in the ODM file, because a statistic and chart must be computed for each of them. The second one is the number of subjects which corresponds to the number of values taken into account for each statistic. The calculation of the mandatory completeness takes a long time, since nearly the entire database is loaded for the calculation. The PDF creation time is only influenced by the number of item tags, since one PDF page is created per item. Since the statistics were already calculated, they are re-used to create the PDF file without any extra calculation effort.

There is nearly no performance difference in executing the program on a local PC or in a Docker container on Linux. The benchmarking results for dataset 3 are even faster when calculated in a Docker instance than the execution in a native environment. This slight difference can probably be explained by different configurations and caching behavior within the Docker environment.

However, the Docker times on a Windows 7 PC are significantly worse, since a Linux virtual machine is been started on Windows 7 and this virtualization increases the RAM access times. This may change with Windows 10 because Microsoft started to officially support Docker and grant access to deeper virtualization techniques within the Windows core [39].

Although it might be counter-intuitive, a runtime improvement cannot be obtained by increasing the available RAM. Even for the fourth dataset the 8 GB Java heap was not used entirely. By outsourcing the clinical data into the database, the memory issues are mainly resolved. However, if the dataset size increases significantly, the size of the in-memory database will also increase and more main memory must be provided to prevent time consuming memory swaps.

### Critical design decisions

#### Performance and stability

Since Java was chosen as programming language and the ODM format is based on XML, the DOM and SAX parsers are the common choices to parse this format [40]. DOM loads the entire XML file into the main memory and creates a tree structure which can then be traversed for further processing. This works well for smaller files but files greater than 40 MB require too much memory for the internal structure. Thus, SAX parser was chosen for implementation of this project because it parses one tag at a time and does not require the complete file to be present in main memory. However, although SAX reduces the amount of memory required for parsing the XML significantly, the number of objects, e.g., over one million clinical data items, are still too much for the Java heap. Thus, the clinical data needed to be stored in a database to reduce the number of Java objects.

#### Data privacy and protection

Data privacy and protection are one of the critical tasks to be taken into account to handle the uploaded clinical data carefully, especially on the web. This is because clinical study data may contain personal information about patients like names, addresses etc. ODM-DA tool uses the in-memory database H2 [30], which does not store the data persistently. This makes standard data persistence and recovery possibilities inapplicable. In our scenario, this is an advantage, because no one can recover deleted data from the database using forensic tools to detect data fragments on the hard disk. Hence, each ODM upload creates a new in-memory database instance which is deleted after the analysis process.

Statistics and additional charts are generated for almost all items. Most chart visualization libraries are API-based third party tools requiring to send even aggregated data to foreign servers, which does not accompany with our data protection claim. For instance the terms of services of Google Charts API does not allow using the libraries offline [41]. Highsoft for example offers Highcharts, an API for a large variety of customizable charts, which can be used online but also on a local server offline [42]. To reduce the number of dependent components JFreeCharts was chosen, which can be integrated easily [28].

#### Repeating elements

ODM supports repeat keys at the level of study events, forms and item groups. This means, for instance on the level of item groups, each item of the group can be completed multiple times in the same form. For example the medication of a patient, if multiple drugs are prescribed. The same applies to the level of forms and study events. Thus, a single subject can contribute several times to the statistics of a single item, which, of course, sophisticates the statistic. Therefore, these cases must be handled with care.

Different potential solutions exist to solve this issue. If multiple numerical values are provided, e.g., for the blood pressure, in a first step the average value per subject could be calculated and only these pre-calculated values could be taken into account for the final statistic. Thus, it is guaranteed that each subject only contributes once per item.

Although this behavior would be suitable for some items, it is not suitable for all of them. If, for instance, the item describes a score, e.g., the EDSS score [43] throughout multiple follow up visits, an increase over time should be recognized, and calculating the average value per patient would result in misleading statistics. In this case, only taking the ‘last’ score into account would probably be the best solution. However, ODM repeat keys are string values, and thereby it cannot be automatically detected which value is related to the last visit.

Another option is to use all values and to ignore the fact that a single subject may contribute the entire data the statistic is based on. Since it is impossible to determine, without additional or user guided information, which behavior is the desired one for each item, we have chosen the last discussed option to use all values and ignore the potential multi-contribution of a subject. The benefit of this approach lies in the consistent handling of all data types, since, e.g., average values do not exist for character based items. However, the presence of repeat keys is indicated in ODM-DA by showing a warn sign at the associated statistic to prompt the user to handle these results with special attention.

### Strengths and weaknesses

The strength of this application is its easy applicability. Anyone can upload an ODM file and get a quick overview of the contained data and its distribution from seconds to less than half an hour (depending on file size and hardware). Also since ODM-DA is open source and simple, statistical questions can be answered without special training and without any costs. During the analysis process, physicians confirmed a better understanding of the structure and content of their study data. The detection of invalid data entries, which was used to improve the overall data quality, was found a useful functionality of ODM-DA by the physicians as well as study personnel. Nearly every uploaded file contained at least one meta item with multiple invalid values, mostly caused by a data type change during the study’s runtime and a not consequent update of already collected data. Also, the completeness measure often showed interesting results and flaws in the CRFs. Although dataset 3 contained over 6000 subjects, only 1300 were actually completed. For dataset 1 every subject was completed since no items were flagged as mandatory. In contrast, dataset 2 had no completed subjects, since each item was flagged mandatory and items like ‘Date of Death’ were empty in 99% of the cases. All this information was obtained by just uploading the ODM files and was not clearly presented in the used clinical studies’ EDCs.

Although the automatic analysis seems to be very easy and efficient, it also has its limitations. Each statistic must remain generic because additional information about the included items is missing. Especially the handling of repeat keys could be improved by an upstream user interface on which decisions about the interpretation could be transacted. Application of medical terminologies or harmonized common data catalogs for clinical studies including their reporting [44–46] or a specific therapeutic area [47] may help to improve the understanding of items fostering an automatic generation of more specific statistics. Overall, ODM Data Analysis cannot replace the complex analytic work of a statistician, but may provide a first impression and good starting point for further analysis.

### Future work

For future work we are planning to work on the overall performance of the tool. Since the bottle neck of the overall performance has been detected in the calculation of the completeness and the population of the database, a significant performance increase should be possible by improving the database structure and shifting more calculations onto the database management system. Furthermore, the support of the entire ODM Standard is planned for future releases. This includes multiple study tags in an ODM file as well as the support of different metadata versions, which are currently ignored.

ODM Data Analysis was designed to generate only generic statistics of each item without the comparison with other items. As a next huge development step the automatic generation of bivariate statistics could be implemented. Giving a quick overview of co-variant items in forms could guide statisticians in the right direction for further data analysis. However, bivariate analysis may increase the runtime significantly and is not canonically performed for variables of different data types. Also the problem of *multiple comparisons* has to be kept in mind.

## Conclusion

In this paper we proposed our open source web-based application for the automatic generation of descriptive statistics for each item of CRFs stored in ODM files. With this tool, physicians are able to extract the often required baseline characteristics of their acquired clinical study data and monitor their completeness without the necessity of learning complex statistical software. Due to its implementation as Docker container and its simple user interface, it can be easily maintained on any standard PC or server with commodity hardware. Although the application cannot replace the complex statistical analysis performed by statisticians, it is a good starting point for a quick overview of the underlying data.

## Supporting information

S1 Video How to export an ODM file from an EDC system. Short screencast how to export data from an EDC system in the required ODM format. OpenClinia is used as example EDC.
(M4V)

S2 Video How to use use ODM Data Analysis. Short screencast how to upload an ODM file and how to navigate through the application.
(M4V)
